# Association Mapping of Diastatic Power in UK Winter and Spring Barley by Exome Sequencing of Phenotypically Contrasting Variety Sets

**DOI:** 10.3389/fpls.2017.01566

**Published:** 2017-09-12

**Authors:** Mark E. Looseley, Micha Bayer, Hazel Bull, Luke Ramsay, William Thomas, Allan Booth, Carla De La Fuente Canto, Jenny Morris, Pete E. Hedley, Joanne Russell

**Affiliations:** ^1^Cell and Molecular Sciences, The James Hutton Institute Dundee, Scotland; ^2^Information and Computational Sciences, The James Hutton Institute Dundee, Scotland

**Keywords:** barley, exome capture, malting quality, diastatic power, QTL mapping

## Abstract

Diastatic Power (DP) is an important quality trait for malt used in adjunct brewing and distilling. Substantial genetic variation for DP exists within UK elite barley cultivars, but breeding progress has been slow due to the limited demand, compared to the overall barley market, and difficulties in assessing DP. Estimates of DP (taken from recommended and national list trials between 1994 and 2012) from a collection of UK elite winter and spring varieties were used to identify contrasting sets of high and low DP varieties. DNA samples were pooled within sets and exome capture sequencing performed. Allele frequency estimates of Single Nucleotide Polymorphisms (SNPs) identified from the sequencing were used to identify genomic locations associated with differences in DP. Individual genotypes were generated from a set of custom KASP assays, both within sets and in a wider germplasm collection, to validate allele frequency estimates and marker associations with DP. QTL identified regions previously linked to variation in DP as well as novel associations. QTL colocalised with a number of genes annotated as having a diastase related function. Results indicate that winter barley is more genetically diverse for genes influencing DP. The marker assays produced by this work represent a resource that is available for immediate use by barley breeders in the production of new high DP varieties.

## Introduction

One of the most economically significant uses of barley (*Hordeum vulgare* L.) worldwide is in the production of alcohol following malting, with about 20% of annual barley production being used for processing (FAOSTAT: http://www.fao.org), the vast majority of which is used by maltsters. During the malting process, endogenous proteolytic and amylolytic enzymes (either present within the mature grain or generated during germination) are released and modify the barley endosperm, with starch being converted into fermentable sugars. The breakdown of starch is catalyzed by a group of enzymes known as diastases and their combined ability to do so is called diastatic power (DP). Whilst most barley varieties can produce malt with sufficient diastase activity to convert all its starch into fermentable sugars during malting and mashing, specific malting applications, such as the use of unmalted grain as an adjunct source of starch in brewing and distilling, require the use of barley malt with higher levels of diastase activity. DP is subject to considerable environmental influence, in particular from differences in nitrogen management regime (Eagles et al., 1995; Chen et al., 2006), but there also exists considerable genetic variation within cultivated barley (Filichkin et al., 2010; Hu et al., 2014). Whilst high DP has been the subject of selection for good malting quality in some regions where adjunct brewing is prevalent, there has been less breeding progress for the character in the UK due to a relative lack of demand for high DP malt. Nevertheless, an increase in the total weight of unmalted wheat used in brewing and distilling has been recorded in recent years in the UK (DEFRA, 2017), and this is due to an increase in the production of grain whisky (Scotch Whisky Association, 2017). The Scotch Whisky Association ceased producing annual production figures for grain whisky after 2012 but the data up to that date can be used to estimate that the amount of wheat used doubled from around 500,000 t in 1980 to just under 1 million t in 2008. This means that the demand for high diastase malt in the UK is around 100,000 t, assuming an inclusion rate of 10%. Allowing for malting losses and the different moisture contents of unmalted and malted barley, this equates to malting purchases in the order of 115,000 t at 85% dry matter. This now represents a significant market, and breeding opportunities exist for new varieties that combine the high agronomic performance associated with modern varieties with high levels of diastase activity. The development of molecular markers that are tightly linked to genes responsible for variation in DP would provide an opportunity for breeders to incorporate this trait into their selection programmes in a cost effective manner.

The major enzymes involved in diastase activity are α-amylase, β-amylase and limit dextrinase (Evans et al., 2010). The activity of these enzymes (particularly β-amylase) has been shown to correlate with DP (Gibson et al., 1995; Georg-Kraemer et al., 2001; Clancy et al., 2003). Starch in the barley endosperm consists of a mixture of amylose and amylopectin. Each is formed of long chains of glucose molecules with (1 → 4) α-glycosidic bonds and, in the case of amylopectin, branches occurring with (1 → 6) α-glycosidic bonds. α-amylase randomly hydrolyses internal (1 → 4) α-glycosidic bonds, whilst β-amylase removes the disaccharide maltose from the non-reducing ends of the resultant dextrins. Finally, limit dextrinase hydrolyses the (1 → 6) α branch in branched dextrins to produce linear dextrins that are accessible for further hydrolysis to maltose by β-amylase. A number of barley genes coding for these hydrolytic enzymes have been mapped. Three β-amylase genes, *Bmy1 Bmy2* and *Bmy3*, are located on chromosomes 4H, 2H, and 4H respectively (Li et al., 2002), and the α-amylase genes *Amy1* and *Amy2* are located on chromosomes 6H and 7H respectively (Knox et al., 1987). The limit dextrinase gene, *Ldx*, was mapped to the short arm of chromosome 7H (Li et al., 1999). In addition to amylase proteins a number of enzyme inhibitors that may also affect DP have been identified in barley, such as barley α-amylase/subtilisin inhibitor (BASI), which inhibits the endogenous barley α-amylase isozyme 2 (AMY2) (Mundy et al., 1983; Nielsen et al., 2004) and limit dextrinase inhibitor (LDI) (Stahl et al., 2004). The genes that code for these proteins have been located on barley chromosomes 2H and 6H respectively (Hejgaard et al., 1984; Karakousis et al., 2003; Stahl et al., 2007). However, surveys of mapped diastase genes have revealed little diversity amongst European and North American cultivated barley (Zhang et al., 2007; Filichkin et al., 2010).

A number of studies have looked at the genetic basis of malting quality traits, including DP as well as α-amylase and β-amylase activity. These have included QTL mapping studies using bi-parental populations (Marquez-Cedillo et al., 2000; Panozzo et al., 2007; Islamovic et al., 2014). Whilst each of these studies sampled a narrow range of genetic diversity, they identified a number of DP QTL across the barley genome. These were generally inconsistent across populations and experiments, suggesting complex genetic control and substantial genotype by environment effects. Other studies have used expression analyses to identify genes that are associated with variation in malting quality parameters (Potokina et al., 2004; Lapitan et al., 2009). Whilst neither of these studies specifically identified structural diastase genes, a number of genes with a putative role in carbohydrate metabolism more generally were associated with variation in DP. Over recent years, high density SNP (single-nucleotide polymorphism) genotyping has allowed the use of genome-wide association scans (GWAS) to survey genetic variation influencing malting quality characteristics in diverse collections (Castro et al., 2013). More recently still advances in genomic technology have provided tools for the generation of high density, population specific markers and genotype data. Enzyme-based genotyping by sequencing (GBS) has been successfully applied in barley (Liu et al., 2014), and exome capture technology has made targeted sequencing of coding regions across the genome a viable tool for mapping quantitative traits with extremely high marker density (Mascher et al., 2013). Such an approach allows the simultaneous detection of SNP markers and mapping of quantitative traits in cultivar collections. Nevertheless, the costs associated with library preparation, sequencing, and subsequent bioinformatics analysis make genotyping large collections expensive using this approach. However, direct sequencing approaches allow allele frequencies from mixed DNA samples to be estimated directly (Gautier et al., 2013). Therefore, an alternative approach is to sequence pooled DNA samples from phenotypically contrasting sets using exome capture in order to identify marker trait associations from the degree of allelic differentiation between pools as a modification of a Bulked Segregant Analysis (BSA) (Michelmore et al., 1991). Sequencing of pooled DNA samples has previously been used to identify candidate genes for binary traits (e.g., Hanson et al., 2007), and for mapping quantitative traits in crop plants (Yang et al., 2015) where it has been termed extreme-phenotype genome wide association study.

The general aim of this study was to test the effectiveness of exome capture and sequencing of pooled genomic DNA as a method for mapping quantitative traits in barley through the accurate estimation (and comparison) of allele frequencies from phenotypically contrasting sets. Specifically, the study aimed to identify QTL and genes responsible for genetic variation in DP in both spring and winter elite UK cultivars, enabling tools for breeders to produce new varieties that are specifically targeted at the production of high DP malt.

## Materials and methods

### QTL mapping

#### Historical data and selection of contrasting sets

DP data for a collection of 592 UK barley varieties (367 spring and 225 winter) was taken from historical recommended (RL) and national list (NL) trial results over the period 1994-2012 as part of the IMPROMALT project (BBSRC: BB/K008188/1). This panel was largely the same as that used in Thomas et al. (2014), with the addition of lines registered on the UK National List up until 2013. Varieties were included in these trials for a variable number of years (minimum 2 and maximum 23) with none present over the whole survey period. In each year, data was collected from between 30 and 53 trial sites and each year, samples from subsets of these sites were micro-malted and the malt analyzed for malting parameters, including DP. Given this structure, Best Linear Unbiased Predictors (BLUPs) of the means of the genotypes were obtained using a mixed model implemented by the REML directive in Genstat 15 (Payne et al., 2011). In this analysis, the random model consisted of year nested within site; trial series (UK Recommended or National List); genotype; the interaction between genotype and year, and the interaction between genotype and site. Variance components derived from this analysis were used to estimate repeatability. In addition, subsets of 100 spring and 100 winter two-rowed barley lines were grown in field trials for harvests 2013 and 2014. Each year, samples from the sites with the highest and lowest grain nitrogen contents were micro-malted and the malt analyzed according to a standard variety testing protocol by member companies from the Maltsters Association of Great Britain as part of the BBSRC Crop Improvement Research Club funded project: “Improving the processability of malting barley” (BBSRC: BB/J019593/1). The four estimates of DP for each genotype from these trials were used to derive an overall mean and combined with the IMPROMALT estimates to derive an overall mean as a BLUP in a REML analysis with a random model consisting of data source (historical vs. processability project) and genotype.

Contrasting sets of high and low DP lines were selected from each of the two germplasm collections (spring and winter barley; Table 1). Each set contained 12 lines. This set size was chosen as a compromise between ensuring that sets represented the extremes of the phenotypic distribution, whilst also ensuring statistical power to detect associations. To maximize diversity and to avoid selecting lines with high genetic similarity within sets, a Euclidean distance matrix was calculated for candidate lines using genotype data from the Illumina barley 9k array iSelect chip (Comadran et al., 2012), using the “dist” function as implemented in “R” (R Core Team, 2014). Lines were sequentially added to sets based on their overall mean score (starting from the most extreme value), but discarded if their distance from an already selected line was less than 20. The spring varieties Belgravia and Olympus were included in the spring high set as they were on the AHDB Recommended List for 2016/17 with the former having a full and the latter a provisional approval for use in grain distilling from the Institute of Brewing and Distilling. To check for the presence of population structure within selected lines, a chi-squared test of homogeneity was conducted for each of the iSelect markers (testing marker allele and set membership), and the quantiles of the observed probabilities, associated with the chi-squared statistics, were compared to the quantiles from the null distribution.

**Table 1:** Varieties selected for inclusion in each of the contrasting sets.

**Spring High DP**	**Spring Low DP**	**Winter High DP**	**Winter Low DP**
Belgravia	Alabama	Acute	Cedar
Chime	Amphora	Alpha	Cypress
JB Maltasia	Brazil	Caption	Diadem
Marthe	Cairn	Concept	Diamond
Monika	Calico	Leonie	Fahrenheit
Olympus	Cindy	Melanie	Marinka
Roxana	NSL 95-1257	Milena	Parasol
Sebastian	Otira	Nectaria	Pedigree
Static	Splash	Pearl	Peridot
Tapestry	Spotlight	Silverstone	Portrait
Turnberry	Vivendi	Sunbeam	Prelude
Westminster	Waltz	Torrent	Tallica

#### Exome capture and sequencing

Seedlings from each of the lines selected for the contrasting sets were grown to the three leaf stage, and 200 mg of leaf material was removed and flash frozen for DNA extraction. Genomic DNA extractions were made using a DNeasy Plant Mini kit (Qiagen) according to the manufacturer's instructions. Extracted DNA was quantified using a Qubit fluorometer (Thermo Fisher) and an equal quantity of genomic DNA was combined for each line in each set (12 lines × 4 sets). Combined DNAs for each sample pool (100 ng each in total) were used for individual exome capture and sequencing. A custom barley exome capture SeqCap EZ library was used throughout, representing approximately 62 Mbp of the barley reference exome (Mascher et al., 2013). DNA library preparation and exome capture was performed using recommended methods in the SeqCap EZ Library SR User's Guide (Nimblegen Roche). Briefly, 100 ng of intact pooled barley genomic DNA was used to generate an Illumina-compatible library using the KAPA Library Preparation Kit as described, utilizing SeqCap adapters with individual indexing to allow downstream multiplexing. Library quality control was established using a Bioanalyzer 2100 (Agilent). Pre-capture amplification, purification with AMPure beads and exome capture was performed as recommended. Following hybridization of each library pool with the barley SeqCap EZ Probe Pool at 47°C for 16 h, beads were washed, and captured DNA recovered. Captured DNA was amplified for each pool using LM-PCR with KAPA HiFi HotStart ReadyMix as described in the protocol, and the final library pools quality checked on a Bioanalyzer 2100 (Agilent). Equimolar amounts of each of the 4 pooled captures were combined and diluted ready for sequencing as recommended. Sequencing was conducted over 2 lanes on an Illumina HiSeq 2500 sequencer to generate 2 × 100 bp paired end reads in Rapid-Run mode.

#### Variant calling and filtering

The reads were mapped to the barley genome reference sequence (Beier et al., 2017; Mascher et al., 2017) using BWA MEM v. 0.7.10 (http://bio-bwa.sourceforge.net/bwa.shtml). The raw SAM output was filtered with the bamtools toolkit (https://github.com/pezmaster31/bamtools) to remove reads that contained more than 4% mismatches, based on their alignment score (AS) flag. Mismatch cut-offs in read mapping are essential for the accuracy of downstream analysis as read mismapping caused by overly relaxed mismatch parameters can lead to dramatically increased false positive rates in variant calling (Ribeiro et al., 2015). Following conversion to BAM format, duplicate reads were removed with Samtools v.1.3.1 (Li et al., 2009) and local realignment of reads around indels performed using the Genome Analysis Toolkit (GATK) (McKenna et al., 2010). The latter adjusts the placement of reads that have been aligned suboptimally around indels, and thereby removes base mismatches that could be misinterpreted as variants in the downstream analysis. Completed mappings were inspected visually using the Tablet assembly viewer (Milne et al., 2010, 2013).

Variant calling was carried out with FreeBayes v0.9.18-3-gb72a21b (Garrison and Marth, 2012) using the parameters: “–haplotype-length 0 –min-alternate-count 5 –min-alternate-fraction 0.05 –min-coverage 10 –pooled-continuous –no-complex –no-mnps –dont-left-align-indels –no-indels –no-population-priors.” BAM files representing high and low DP pools were merged separately for the spring and winter lines prior to variant calling.

Identified variants were selected according to a number of quality characteristics. Variants with a phred score less than 50 were excluded along with variants that were fixed for the same (non-reference) allele in each contrasting set. Markers were considered to be fixed if they had a reference allele frequency (RAF) less than 0.01 in either set. In addition, only variants with at least 100 reads in each set were included. This filter was applied to limit the effect of sampling error in the estimates of allele frequencies.

#### Identification of associations

Allele frequency estimates for each marker within each set were calculated directly from read numbers of each allele. Allele frequency differences (AFD) for each marker were calculated as the reference allele frequency of the high DP set minus the reference allele frequency of the low DP set, giving a possible range between −1 and 1. Genomic locations with an absolute estimated allele frequency difference greater than 0.75 were chosen as putatively associated loci. Whilst it was not possible to derive a null distribution for allele frequency differences (which depends on the allele frequencies in the population from which the sets were drawn), such a difference corresponds to a minimum −log10(p) of approximately 3.8 (when allele frequencies in the original population are equal and allele frequency estimates are perfect).

Specific winter and spring barley associations were identified independently from either the winter or spring contrasting sets. QTL were considered independent if associated markers were separated by at least 400 Mbp in centromeric regions, or 10 Mbp in non-centromeric regions (corresponding, very approximately, to 10 cM in each case) (Mascher et al., 2017) in which there was no other marker with an AFD > 0.75. This threshold was chosen to minimize the possibility of selecting single QTL effects multiple times.

### Identification of diastase genes from reference genome

Annotations of high confidence genes from across the Morex reference genome (Mascher et al., 2017) were searched for terms related to diastase activity. These included PFAM annotations containing: PF00128 (Alpha amylase, catalytic domain); PF01356 (Alpha amylase inhibitor); PF01373 (Glycosyl hydrolase family 14); PF02806 (Alpha amylase, C-terminal all-beta domain) or; PF16657 (Maltogenic amylase, C-terminal domain). In addition, genes with functional descriptions (taken from Mascher et al. (2017)) containing: “amylase” or “dextrin” were also selected.

### Design of KASP assays

In order to validate associations identified from the sequence data, SNPs were converted to KASP markers to allow for cost-effective individual genotyping of a limited number of candidate loci (Semagn et al., 2014). Flanking sequences from associated markers identified from the exome capture analysis were extracted from the Morex reference genome and used to design KASP assays. These included at least one marker from each QTL location (the most highly differentiated) as well as differentiated SNPs from putative diastase genes collocated with identified QTL (i.e., within the criteria for independent QTL described above). If no SNP with an allele frequency difference greater than 0.75 was present within the putative diastase gene, the criterion was relaxed to an absolute difference of greater than 0.6. KASP assays were designed from sequence flanking the SNP (extracted from the Morex genome sequence) and supplied by LGC Genomics.

### Validation of allele frequency estimates

#### KASP assays

In order to verify allele frequency estimates derived from the exome capture sequencing analysis, each of the varieties selected for inclusion in the contrasting sets was individually genotyped for each of the KASP assays using an Applied Biosystems StepOne Plus Real-Time PCR system according to the manufacturer's instructions. The reaction mix was held at 37°C for 1 min for the pre-PCR read. Subsequently it was held at 94°C for 15 min prior to 10 cycles of 20 s at 94°C and 1 min at 62°C. A further 32 cycles of 20 s at 94°C and 1 min at 55°C were performed prior to a 1 min hold at 37°C for the post-PCR read. Automated clustering was performed using the StepOne Software v2.3 and checked by eye. A chi-squared test was used to test homogeneity of marker genotypes within each set.

#### iSelect allele frequencies

Genotype data from the Illumina barley 9k iSelect chip (Comadran et al., 2012) were available for each of the selected lines. Physical positions of iSelect markers were identified on the Morex reference genome by BLAST searching SNP manifest sequences against the total pseudomolecule sequence of 4.8 Gbp (Mascher et al., 2017). This yielded unambiguous positions for 4,986 9k iSelect markers. These were compared with exome capture variants to identify overlaps between the two sets of SNPs. This allowed estimated reference allele frequencies to be compared with the true reference allele frequency.

### Validation of associations

In order to test the robustness of the marker associations identified from the exome capture sequencing, a wider set of varieties (comprising 82 spring and 76 winter varieties from the original cultivar set) was genotyped. Details of all the lines used for this genotyping are given in Supplementary Table 1. A *t*-test was used to test for differences in the historical DP estimates of lines carrying either the high or low allele of candidate markers.

## Results

### Selection of contrasting sets

On average there were 25 sites across both seasonal growth habits with DP data for each year for the 592 lines in the historical data set. Repeatability was moderately high for DP for each growth habit (0.53 in springs and 0.61 in winters). In addition, DP estimates based on 2 years of field trial data/ micro-malting were taken from 100 spring and 100 winter varieties but the structure of this experiment did not permit a true estimate of the repeatability across the whole data set. There was considerable overlap between the lines for each data source so the total number of lines for which data was available was 602 (371 spring and 231 winter barley lines), from which an overall mean was derived by a REML analysis (Supplementary Table 1). Averaging them for each seasonal growth habit, the mean DP was similar in both (93.6 in spring types compared to 93.8 in winters) but with a lower standard deviation in the springs (12.9 compared to 17.1).

**Figure 1:**
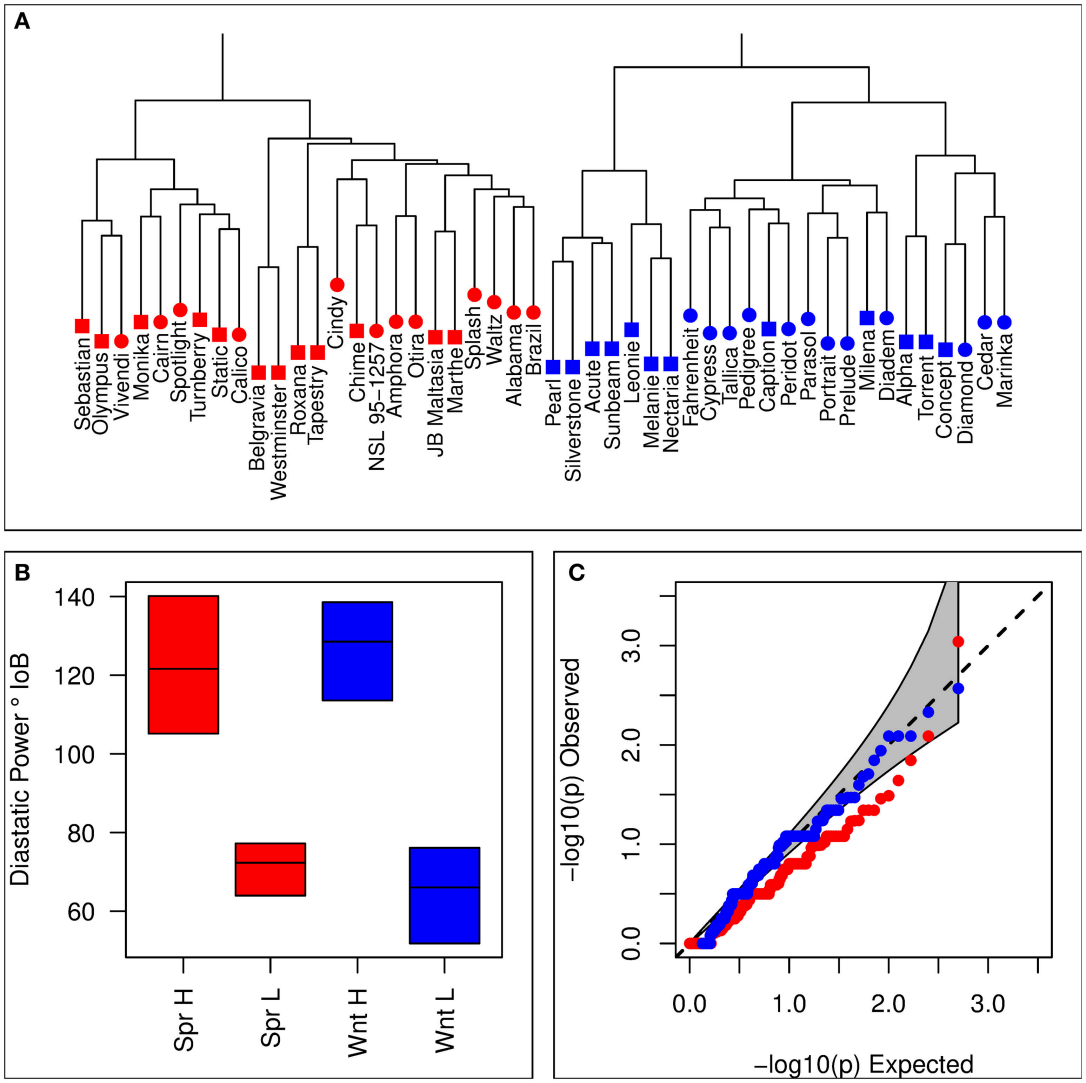
Summary of lines selected for inclusion in contrasting sets. **(A)** Hierarchical clustering based on the distance matrix of selected lines. Blue symbols represent winter varieties, and red symbols represent spring lines. Square symbols indicate membership of a high DP set, and circular symbols a low DP set. **(B)** Distributions of estimated diastatic power for each of the four contrasting sets. Horizontal lines indicate within set means, and colored boxes show the range. **(C)** Observed quantiles of the *p*-values for a chi-squared test of homogeneity (marker genotype against set membership) for iSelect 9k marker genotypes of selected lines. Red symbols represent the spring contrast, and blue symbols the winter contrast. The gray region represents the 95% confidence interval for expected values.

Sets of 12 lines each for winter and spring contrasting sets were selected based on the combined estimate of DP from both historical estimates and the means taken from the processability project. 9k iSelect genotypes were used to avoid selecting highly genetically similar varieties. Means from each of these data sources were generally in agreement, with moderate positive correlations between historical estimates and each of the trials for the processability project (Supplementary Table 1). Details of barley varieties selected for each set are given in Table 1. There was no evidence that selection based on phenotype was associated with population structure, with hierarchical clustering analysis showing no tendency for clustering by set (Figure 1A), and no excess significance in the chi-squared test of association between iSelect genotypes and set membership compared to the expected values (Figure 1C). Selected lines formed clearly differentiated phenotypically contrasting sets in both winter and spring types (Figure 1B).

### Identification of diastase related genes

**Figure 2:**
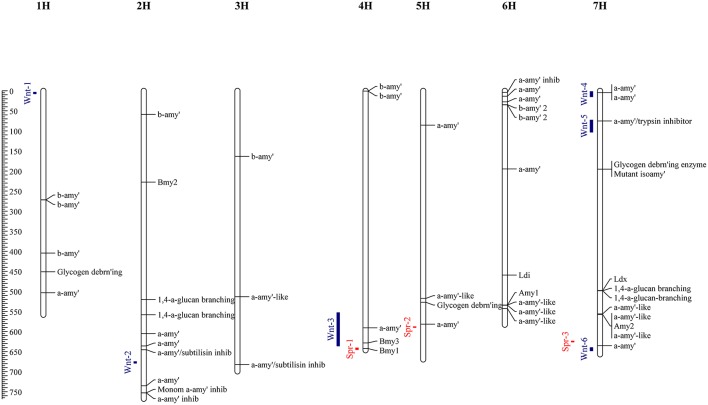
Physical map locations (Mbp) of QTL identified by allele frequency differences between high and low DP sets. Associations identified from the spring set are indicated by red bars, and the winter set as blue bars. Genes identified (by homology) from the barley reference sequence with putative (or known) diastase function are indicated along with functional descriptions or gene names for known barley diastase genes.

To supplement the mapping results, the barley reference genome was searched in order to identify genes which may play a role in DP, and thus place these in the context of identified QTL causing variation in diastase activity. Homology based searching of the Morex high confidence genes identified 54 with putative diastase function; these comprised 24 genes with functional descriptions suggesting α-amylase structural genes; 8 α-amylase inhibitors; 12 β-Amylase structural genes; 3 Glycogen debranching enzymes, and 7 1,4-alpha-glucan-branching enzyme (Figure 2). Full details of these genes are given in Supplementary Table 2.

### Exome capture and sequencing

The Illumina HiSeq sequencing run generated approximately 720 M reads, with read numbers for the four pools (high versus low DP × winter vs. spring lines) varying between ~170 M and 185 M reads (Table 2). This equates to approximately 275- to 300-fold coverage of the 61.6 Mbp exome capture array per pool sequenced. All sequence data is available at the European Nucleotide Archive (Accession: PRJEB21308).

**Table 2:** Summary of read numbers from each of the three sequenced sets.

**Season pool**	**DP**	**# Reads**
spring	high	181,564,086
spring	low	185,305,764
winter	high	183,044,728
winter	low	170,457,518
Total		720,372,096

### Variant calling and filtering

Variant calling was conducted using FreeBayes software. Following mapping and duplicate read removal, a total of 362,398,706 reads remained for further analysis. The winter and spring contrasting sets were analyzed independently. Prior to filtering, this procedure identified 859,817 variants in the winter sets. Of these 782,766 had a phred score greater than 50, and 546,983 were also polymorphic within the exome capture sequences (i.e., not simply fixed differences between lines in the contrasting set and Morex). A final filtering step to exclude SNPs with fewer than 100 reads left a final set of 77,523 SNPs. For the spring set the corresponding numbers were: 859,511 variants, of which 778,676 had a phred score greater than 50; 526,234 were polymorphic, and 83,568 had more than 100x read coverage.

### Identification of associations

Allele frequency estimates from the filtered SNPs were made from the read counts of each allele from each set. These were used to identify markers with highly differentiated frequencies between high and low sets. In the winter sets, 66 markers with an absolute allele frequency difference greater than 0.75 were identified, corresponding to 6 distinct physical positions. These QTL (Wnt-1-6) were located on chromosomes 1H, 2H, 4H, and 7H (Figure 2, Table 3). In the spring sets 32 markers showed an allele frequency difference greater than 0.75, corresponding to 3 distinct physical locations (QTL Spr-1-3) on chromosomes 4H, 5H, and 7H (Figure 2, Table 3). For each QTL, the marker that showed the peak AFD was chosen as representing the QTL and all except Wnt-2, Spr-2 and Spr-3 were supported by at least 5 differentiated markers (Table 3). Wnt-3, Spr-1, Spr-2, Wnt-4, Wnt-5, and Wnt-6 were all QTL intervals that either contained (or were close to) known diastase genes, or genes which were annotated with a putative diastase function (Figure 2).

**Table 3:** Summary of putative QTL locations based on allele frequency difference (AFD) between phenotypically contrasting sets.

**Name**	**Type**	**Chromosome**	**Interval (bp)**	**Markers**	**Peak (bp)**	**Max AFD**
Wnt-1	Winter	1H	3,667,293–7,425,992	22	4,190,695	0.92
Wnt-2	Winter	2H	–	1	677,198,003	0.80
Wnt-3	Winter	4H	553,588,912–636,354,086	5	636,354,086	0.79
Wnt-4	Winter	7H	1,776,584–15,062,551	12	4,498,971	0.89
Wnt-5	Winter	7H	73,549,032–103,711,832	10	101,614,577	0.79
Wnt-6	Winter	7H	640,005,292–647,798,125	13	647,757,050	0.92
Spr-1	Spring	4H	641,153,957–645,128,458	29	644,846,286	0.98
Spr-2	Spring	5H	–	1	589,149,449	0.76
Spr-3	Spring	7H	625,333,885–625,333,886	2	625,333,885	0.76

*QTL names are shown along with the set (winter or spring) in which they were detected. Chromosome locations, along with the intervals in which marker differentiation was detected (indicated as base pair positions on the reference sequence pseudomolecules), are shown along with the number of markers within each QTL interval. The base pair positions and AFDs (high set–low set) of the most differentiated marker is shown for each QTL*.

### Validation of allele frequency estimates

#### iSelect

**Figure 3:**
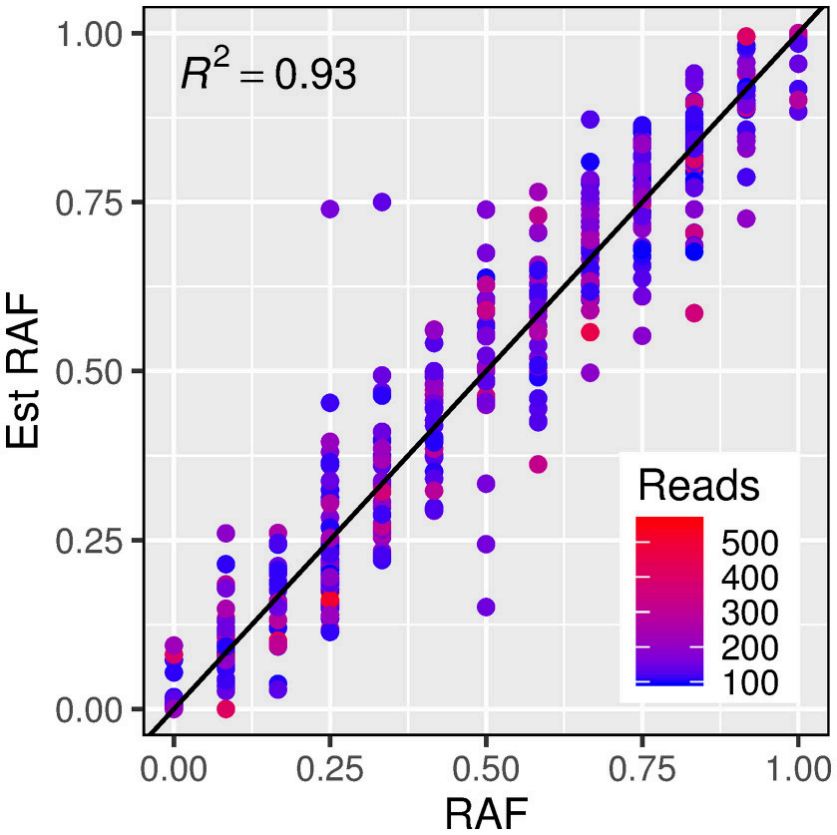
Relationship between estimated reference allele frequencies (y axis) and true reference allele frequency (x axis) from exome capture reads and iSelect 9k genotypes respectively. The dashed line represents a “one to one” linear relationship and the *R*^2^-value indicates the proportion of the variance in the estimated allele frequency accounted for by this model. The color of the points indicates the read count for each estimated frequency.

In order to test the robustness of the estimates of allele frequencies made from exome capture sequencing, a subset of exome capture SNPs that overlapped with those present on the iSelect 9k chip (and thus had known allele frequency in each set) was identified. A total of 4,986 iSelect SNP markers could be mapped unambiguously to the Morex reference sequence. Of these, 460 unique SNP markers (with 379 from the winter set, and 323 from the spring set) were also identified as variants in the exome capture set. A linear model describing a one to one relationship between estimated and true reference allele frequencies accounted for a high proportion of the variance in estimated allele frequencies (Figure 3, *R*^2^ = 0.93).

#### KASP genotyping

A set of 13 SNPs, identified as having a putative association with DP, were converted into KASP assays. As described above, this set included at least one marker from each QTL location as well as differentiated SNPs from putative diastase genes, (where these were collocated with the identified QTL) using a more relaxed differentiation threshold. Individual genotyping of the lines selected for the contrasting sets showed a strong correlation between absolute values of the estimated allele frequency differences from the exome capture and sequencing and absolute values of the true AFD (*r* = 0.67) (Table 4). Individual genotyping indicated that most of the markers showed strong evidence against homogeneity between sets, with marker alleles showing clear associations with one or other set (Table 4). In almost all cases, the absolute AFD was lower than the estimated value (Table 4).

**Table 4:** Details of KASP assays designed from marker associations identified from exome capture sequencing.

**Marker**	**Type (QTL)**	**Chromosome**	**Position (Mb)**	**Gene**	**Functional desctription**	**Est AFD**	**True AFD**	**χ^2^**	**−log10p**
SNP Assay 1	Winter (Wnt-1)	chr1H	4,190,695	HORVU1Hr1G001960	12-oxophytodienoate reductase 2	0.92	0.71	14.29	3.80
SNP Assay 2	Winter (Wnt-2)	chr2H	677,198,003	HORVU2Hr1G096960	glutathione peroxidase 6	0.80	0.40	3.2	1.13
SNP Assay 3	Winter (Wnt-3)	chr4H	636,354,086	HORVU4Hr1G087230	Ectonucleoside triphosphate diphosphohydrolase 5	0.79	0.60	10.8	2.99
SNP Assay 4	Winter (Wnt-4)	chr7H	4,498,971	HORVU7Hr1G002370	Glutathione S-transferase family protein	0.89	0.57	9.14	2.60
SNP Assay 5	Winter (Wnt-4)	chr7H	15,062,551	HORVU7Hr1G010690	Acid phosphatase 1	0.80	0.82	14.73	3.91
SNP Assay 6	Winter (Wnt-5)	chr7H	74,643,257	HORVU7Hr1G034860	Unknown protein; BEST Arabidopsis thaliana protein match is: unknown protein.	0.78	0.85	18.62	4.80
SNP Assay 7	Winter (Wnt-5)	chr7H	75,226,930	HORVU7Hr1G035020	Alpha-amylase/trypsin inhibitor	0.78	0.80	12.8	3.46
SNP Assay 8	Winter (Wnt-6)	chr7H	647,757,050	–	-	0.92	0.50	8	2.33
SNP Assay 9	Spring (Spr-1)	chr4H	642,563,684	HORVU4Hr1G089510 *(Bmy1)*	beta-amylase 5	0.55	0.06	0.12	0.144
SNP Assay 10	Spring (Spr-1)	chr4H	644,846,286	HORVU4Hr1G090300	Transcriptional coactivator/pterin dehydratase	0.98	0.85	18.62	4.80
SNP Assay 11	Spring (Spr-2)	chr5H	589,149,449	HORVU5Hr1G092740	unknown function	0.76	0.80	12.8	3.46
SNP Assay 12	Spring (Spr-3)	chr7H	625,333,886	–	–	0.75	0.33	4	1.34
SNP Assay 13	Spring (Spr-3)	chr7H	635,239,502	HORVU7Hr1G112360	Alpha-amylase	0.61	0.14	0.29	0.23

*The population from which the association was identified (along with the QTL) is indicated along with its chromosome and physical location, and the name and functional description of the gene containing the SNP. Estimated absolute allele frequencies (High DP set–Low DP set) from the exome capture and sequencing are shown along with the true allele frequency (from individual genotyping). Results from a chi-squared test of homogeneity are also shown*.

### Validation of associations

**Figure 4:**
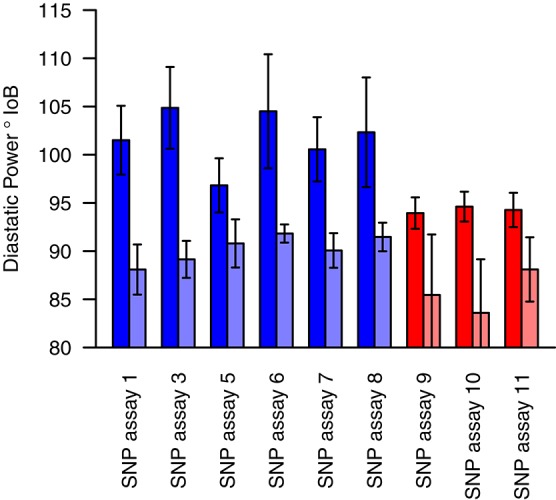
Mean diastatic power of varieties carrying either the high (dark shades) or low (light shades) allele at each of the candidate markers. Blue bars indicate SNPs identified from the winter sets, and red bars those from the spring sets. Error bars indicate the standard error in the mean.

Markers for which the allele frequency contrasts between sets were validated in the individual genotyping were used to genotype a panel of varieties (78 for winter sets and 85 for spring sets), with nine validated from the 19 markers tested in total. For each marker tested, varieties carrying the high allele (as identified in the contrasting sets) had a higher estimated DP than varieties carrying the low allele. In most cases, this difference was highly significant (*p* < 0.001), with the exception of SNP assay 5 (Wnt-4), SNP assay 9 (Spr-1) and SNP assay 11 (Spr-2) (Figure 4, Table 5). In the case of SNP assay 9, this is consistent with the lack of differentiation when true AFDs were calculated.

**Table 5:** Mean DP estimates for varieties with either high or low alleles at candidate markers.

**Marker**	**Type**	**Allele**	**Count**	**Mean (SEM)**	***t***	**df**	***p***
SNP Assay 1	Winter	H (T)	33	101.5 (3.57)	3.23	74	<0.001
		L (G)	43	88.1 (2.6)			
SNP Assay 3	Winter	H (T)	24	104.9 (4.24)	4.05	73	<0.001
		L (A)	51	89.1 (1.92)			
SNP Assay 5	Winter	H (C)	36	96.8 (2.81)	1.67	73	0.05
		L (T)	39	90.8 (2.5)			
SNP Assay 6	Winter	H (A)	12	104.5 (5.91)	4.07	73	<0.001
		L (T)	63	91.8 (0.94)			
SNP Assay 7	Winter	H (C)	28	100.6 (3.34)	3.15	74	0.001
		L (G)	48	90.1 (1.8)			
SNP Assay 8	Winter	H (A)	16	102.3 (5.69)	2.75	74	0.004
		L (G)	60	91.5 (1.49)			
SNP Assay 9	Spring	H (T)	65	93.9 (1.63)	1.9	80	0.031
		L (G)	17	85.5 (6.28)			
SNP Assay 10	Spring	H (T)	64	94.6 (1.55)	2.68	80	0.005
		L (G)	18	83.6 (5.55)			
SNP Assay 11	Spring	H (A)	53	94.3 (1.78)	1.8	79	0.038
		L (G)	28	88.1 (3.34)			

*The nucleotide associated with each allele is shown in brackets. For each marker, the number of varieties in each allelic category is shown, and the mean for each category (with standard error of the mean shown in brackets). The t-statistic for the comparison of the two alleles is shown also, along with the p-value for the right tail of the t-distribution (testing the hypothesis that the mean of the high allele is greater than that of the low allele). Variation in the total number of lines between markers is due to missing data*.

## Discussion

The results presented here demonstrate the effectiveness of using allele frequency estimates from pooled NGS data from phenotypically contrasting sets to map quantitative traits, allowing the use of extremely high marker density along with maximum phenotypic diversity. The approach has a number of limitations compared to a standard GWAS approach, specifically the inability to estimate marker effects from the initial sequence data, and the inability to calculate the statistical significance of the test statistic without also estimating allele frequencies for the original cultivar collection (e.g., Yang et al., 2015). Nevertheless, this approach was used to identify genomic regions (and candidate genes) associated with variation in a complex malting quality trait, and associations were confirmed by genotyping using a separate set of lines.

### Diastase genes and associations

A number of putative associations were identified from the exome capture and sequencing. The majority of the associated loci identified were from winter barley, suggesting that this germplasm set is more diverse for genes influencing DP. This is consistent with a higher genetic diversity in winter germplasm in general (Thomas et al., 2014), but may also reflect stronger historical selection for malting quality traits in spring barley, effectively resulting in the near fixation at malting quality loci. This hypothesis is supported by the observation of higher variability in DP estimates seen in winter varieties compared to springs.

The availability of full sequences for each of the barley chromosomes, now allows QTL regions to easily be placed in the context of the underlying gene content. Homology based searching of the *H. vulgare* reference genome identified a number of high confidence genes that may be diastase related. These included a number of α- and β-amylases located in genomic regions not previously reported to be associated with diastase activity. The majority of QTL identified aligned with genes annotated as diastase related. Whilst this study does not present evidence that demonstrates that these genes are causal to the identified QTL, the correspondence between these and the positions of the QTL provides a set of candidate genes, as well as offering further support to the identification of these regions as QTL influencing DP. The generation of individual sequence data from high and low DP varieties across these loci (rather than the sequences from pooled samples that were generated in this study) will help to address whether variation at these genes is responsible for differences in DP seen within the cultivar collection.

In both winter and spring contrasts, associations were detected in the telomeric region of chromosome 4HL. This region has previously been associated with QTL for DP and β–amylase activity and contains two known β–amylase genes (*Bmy1* & *Bmy3*). Previous studies have linked variation at the *Bmy1* locus with variation in DP (Hayes et al., 1997; Coventry et al., 2003). Results presented in this study suggest that an additional α-amylase gene (*HORVU4Hr1G073630*) may also be present in this region, although no differentiated SNPs were identified in this gene from the sequence data generated in this project. Variation at these genes may be associated with differences in DP in both spring and winter barley and therefore represent good candidates for detailed characterization. However, the ability of bi-parental QTL mapping studies to distinguish between these linked loci is low due to limited recombination during the generation of mapping populations (Zhu et al., 2008). The improved reduction of linkage disequilibrium for genome-wide approaches, when combined with exome capture sequencing and the new genome sequence, offer the potential to increase the resolution of mapped QTL. Indeed, the results presented here suggest that variation at the *Bmy1* locus is unlikely to be responsible for the QTL on chromosome 4H in winter varieties, but is more likely to be responsible for the QTL seen in the spring sets (the winter QTL being telomeric to the physical position of *Bmy1*). Nevertheless, the SNP marker designed in the *Bmy1* gene (SNP Assay 9) was not as highly associated with DP as a closely linked marker (SNP Assay 10). This observation could either be interpreted as reflecting a gene (linked to *Bmy1*) influencing the trait, or being caused by genetic diversity within a linkage block containing both markers, causing a lower correlation between the causal variant and *Bmy1* SNP. Given the small number of candidate markers being considered, it is not possible to distinguish between these explanations given the current data. There are 97 reported high confidence gene models reported across the QTL interval (Mascher et al., 2017), some of which are likely to influence carbohydrate metabolism. Detailed characterisation of each of the candidate genes in the variety collection may help to address the specific genetic control of DP across this region of chromosome 4H, which would be of considerable use for breeders in both the selection of parental lines for crosses, as well as in marker assisted selection.

The majority of QTL identified collocate with known (or putative) structural diastase genes. One of the candidate QTL (at 75 Mbp on chromosome 7H) is linked to a gene encoding a putative α-amylase inhibitor (*HORVU7Hr1G035020*), suggesting that genetic variation at endogenous amylase inhibitor loci may also influence malt diastase activity. Indeed, a SNP assay designed in this gene (SNP Assay 7) showed a stronger association with DP in the wider variety collection than the peak marker identified from the exome capture data (SNP Assay 6). Due to this, the gene represents an extremely strong candidate for follow-up studies. Variation in expression of an α-amylase/subtilisin inhibitor has previously been described as correlating with β-amylase activity (Potokina et al., 2004), but the expressed sequence tag (EST) identified by that study (HY06J10V) maps to the telomeric region of chromosome 2H on the Morex reference assembly, distal to the QTL identified from the winter sets in this work and is located in gene *HORVU2Hr1G090750* (Figure 3; Supplementary Table 2).

One of the strongest associations identified in the current study was identified in the telomeric region of the short arm of chromosome 1H. This is potentially collocated with a QTL reported in a contrast between Australian and Canadian malting barleys (Zhou et al., 2016). The 3.8 Mbp interval identified in the current study contains 121 high confidence genes (Mascher et al., 2017). Whilst none of these are annotated as having a putative diastase function, a large number are annotated as having a role in protein or carbohydrate metabolism. Detailed characterization of this region will be required to identify candidate genes underlying this QTL effect.

The putative QTL effect on chromosome 2H identified in the winter sets were only supported by one differentiated marker, and individual genotyping of the selected lines showed that this was likely to be an overestimation and thus could represent a spurious association. This illustrates the importance of identifying multiple differentiated markers to avoid spurious associations when using estimates of allele frequencies for association mapping.

### Allele frequency estimates

Central to the mapping method employed in this study is the ability to accurately estimate allele frequencies from next generation sequencing of pooled DNA samples. A number of potential sources of error might influence estimates of allele frequencies, including uneven contribution of individuals, preferential capture of alleles, or sampling errors when read coverage is low (Gautier et al., 2013). Whilst experimental sources of error (such as uneven contribution of individual lines to pooled samples) can only be controlled by careful laboratory technique, error associated with read sampling can be adjusted during analysis. Comparison between exome capture variant calling and known genotype data from the Illumina barley 9k iSelect chip indicate that the adoption of a 100x read coverage threshold provided a data set with highly accurate estimates of allele frequencies. In addition, individual genotyping of lines from each contrasting set confirmed that the majority of the markers that were identified as being highly differentiated from pooled exome capture reads were genuinely differentiated between high and low sets. Whilst AFD values were lower than the estimates for almost all of the KASP markers, this is almost certainly due to regression to the mean following the selection of extreme AFD estimates and does not indicate a bias in allele frequency estimates. This suggests that, when appropriate filtering of variant calls is conducted, allele frequency estimates from mixed sample NGS data sets offer the ability to accurately identify differences in allele frequency between combined DNA samples. This allows considerable savings in cost, as well as the ability to sample higher numbers of lines than would be possible if individual libraries had to be prepared for each individual.

### Validation of associations

A further question addressed by this study was whether differentiation between phenotypically contrasting sets was an effective method for identifying genuine marker-trait associations. This is particularly important as associations were identified from absolute differences in allele frequency. Therefore, the significance of such a difference was not possible to calculate; the null distribution being dependent on the unknown allele frequencies in the original cultivar collection. To address this, associations were tested by genotyping in a wider germplasm collection in order to allow an independent validation of candidate markers. This analysis showed that significant differences (in historical DP estimates) were present between lines carrying alternative alleles at candidate markers. This result confirms that the QTL detection methodology used in the study is an effective method for identifying genuine marker associations with quantitative traits, irrespective of their genetic architecture. This can be applied to historical or *de novo* phenotypic data and therefore has wider application as a cost-effective method for employing NGS approaches as a means to conduct association analyses.

## Conclusion

This study demonstrates that the use of NGS techniques on pooled DNA from phenotypically contrasting sets represents a powerful method for conducting GWAS studies at extremely high marker density. This can directly lead to the identification of candidate genes for quantitative phenotypic traits, provided that appropriate methods are used for controlling against population structure and spurious associations from experimental sources of error. Applying this to spring and winter cultivar collections, has led to the identification of at least six novel QTL for the genetic control of DP in UK barley and provided candidate genes that can be followed up in subsequent studies.

## Author contributions

ML contributed to experimental design, analyzed and interpreted data, and wrote the manuscript. MB advised on experimental design, conducted bioinformatics analysis and contributed to manuscript preparation. HB contributed to the analysis of historical data, coordinated preparation of variety collections for sequencing and genotyping and edited the manuscript. LR contributed to the experimental design and edited the manuscript. WT contributed to the experimental design, analyzed historical malting quality data and contributed to manuscript preparation. AB conducted genotyping and preparation of DNA for sequencing. CD conducted genotyping. JM prepared libraries for sequencing. PH advised on experimental design, coordinated exome capture and sequencing, and contributed to manuscript preparation. JR designed the experimental approach, coordinated the research and contributed to manuscript preparation.

### Conflict of interest statement

The authors declare that the research was conducted in the absence of any commercial or financial relationships that could be construed as a potential conflict of interest.
